# Psychodermatological Disorders in Patients With Primary Psychiatric Conditions: Cross-Sectional Study

**DOI:** 10.2196/47769

**Published:** 2023-10-02

**Authors:** Atinuke Arinola Ajani, Fatai Olatunde Olanrewaju, Olumayowa Abimbola Oninla, Olanrewaju Ibigbami, Samuel Kolawole Mosaku, Olaniyi Emmanuel Onayemi, Olayinka Olasode

**Affiliations:** 1 Department of Dermatology and Venereology, Obafemi Awolowo University Ile Ife Nigeria

**Keywords:** affective disorders, anxiety, stress-related, and somatoform disorders, primary dermatological disorders with psychiatric co-morbidity, primary psychiatric disorders with dermatologic manifestations, primary psychiatric psychodermatoses, psychocutaneous disorders, psychophysiological disorders, schizophrenia

## Abstract

**Background:**

Psychodermatological disorders (PDs) and their associations with mental health problems are one of the most frequent research themes in dermatology outpatient settings. Surprisingly, very few studies have been conducted to evaluate PDs among patients with primary psychiatric conditions. As such, the relationship between preexisting psychiatric conditions and comorbid PDs is underrepresented in the literature.

**Objective:**

This study examined the prevalence and distribution of PDs among adults with primary psychiatric conditions and determined their association with underlying psychiatric diagnoses.

**Methods:**

We conducted a cross-sectional analysis at a tertiary health care facility in southwestern Nigeria. Comorbid PDs were identified and classified using preexisting classification systems. A bivariate analysis was conducted to determine the association between PDs and underlying psychiatric conditions. The level of statistical significance was set at *P*<.05.

**Results:**

The study included 107 patients with mental health disorders, of whom 64 (59.8%) were female. The mean age of the patients was 40.73 (SD 13.08) years. A total of 75 (75/107, 70%) patients had at least one comorbid PD. The prevalence of PDs was highest in patients with affective disorders (15/20, 75%) and least in those with schizophrenia (45/66, 68%). PDs associated with delusions or hallucinations and somatoform symptoms were 9 and 13 times more frequent in patients with anxiety disorders compared to those with other psychiatric conditions (*P*=.01; odds ratio [OR] 9.88, 95% CI 1.67-58.34 and *P*=.003; OR 13.13, 95% CI 2.34-73.65), respectively. In contrast, patients with schizophrenia were significantly less likely to be diagnosed with dermatoses resulting from delusions or hallucinations (*P*=.002; OR 0.04, 95% CI 0.00-0.75). A weak but significant negative association was also found between psychophysiological PDs and anxiety disorders (ϕ=–0.236; *P*=.02).

**Conclusions:**

This study provides important insights into the overwhelming burden of psychodermatological conditions in patients with mental health disorders and specific associations with underlying psychiatric diagnosis.

## Introduction

Psychodermatological disorders (PDs) are a heterogeneous group of skin conditions that are significantly associated with mental disorders. Dermatoses that fall into this category often have a significant negative impact on mental health or may also run a clinical course that is determined by the primary psychiatric or psychological disorder. On one hand, skin conditions can precipitate serious psychiatric illness in individuals without preexisting mental health problems [1,2], while on the other hand, primary psychiatric conditions such as trichotillomania present primarily with skin complaints. In the latter scenario, the skin conditions may be the sole manifestation of the underlying psychiatric conditions, and the patient may present first to the dermatologist. Very often, however, the relationship between skin conditions and mental health is bidirectional, complex, and incompletely understood [1,3,4]. A vicious cycle in which psychological factors trigger or aggravate skin conditions and the exacerbated dermatoses induce further psychological problems is a characteristic finding of typical PDs.

Many studies have shown that the burden of psychological and psychiatric problems among patients with dermatological conditions is high and that there are significant relationships between anxiety, depression, and various PDs that have a serious impact on therapeutic results [5-9]. While the majority of research has focused on determining the link between primary skin conditions and the psychiatric or psychological factors that exacerbate or complicate them, very few studies have examined the connection between preexisting psychiatric conditions and comorbid PDs [10,11]. Consequently, existing data on PDs frequently demonstrate a bias toward primary dermatological disorders with psychiatric comorbidities (PDDPCs), while other PDs are underrepresented and their relationships with major psychiatric conditions are underresearched.

The aim of this study was to explore psychodermatological conditions among a sample of patients with mental health disorders receiving care at a mental health clinic in southwestern Nigeria. The specific objectives were to (1) determine the prevalence and pattern of PDs in patients with primary psychiatric conditions and (2) determine the sociodemographic and illness-related factors that are associated with PDs among the study population.

## Methods

### Overview

This was a cross-sectional hospital-based study conducted among patients with primary psychiatric conditions receiving care at the outpatient mental health clinics of the Obafemi Awolowo University Teaching Hospitals Complex in southwestern Nigeria.

### Recruitment and Data Collection

Adults with primary psychiatric conditions diagnosed using Diagnostic and Statistical Manual of Mental Disorders, 4th edition (DSM-IV) criteria by an experienced psychiatrist were recruited consecutively between January and April 2017. Psychiatric diagnoses were categorized into three main groups, as follows: (1) the affective disorders group, comprising patients with major depressive disorders, those with bipolar affective disorders, and those with manic episodes; (2) the anxiety and somatoform disorder group, comprising patients diagnosed with generalized anxiety disorders, those with unspecified anxiety disorders, those with social anxiety disorders, and those with somatoform disorders; and (3) the schizophrenia group, under which patients with either schizophrenia or schizotypal disorders were grouped.

**Figure 1:**
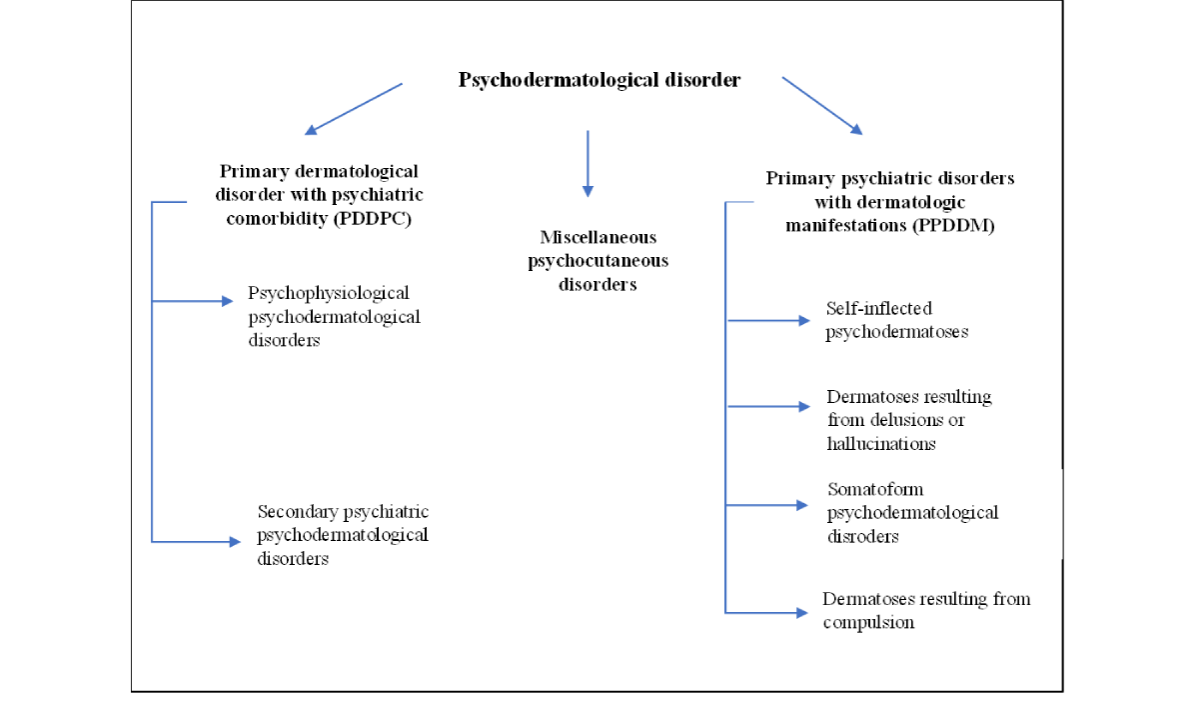
Algorithm for the classification of psychodermatological disorders.

Skin conditions were classified as either psychodermatological or nonpsychodermatological. Psychodermatological conditions, which comprised dermatoses that have significant associations with mental health, were further separated into 3 main categories guided by underlying pathophysiologic mechanisms and preexisting classification systems [12-16]. These include PDDPCs, primary psychiatric disorders with dermatologic manifestations (PPDDM), and miscellaneous PDs [12-15]. The classification system proposed by Koo and Lee [17] was adapted to further classify the PDDPCs into psychophysiological and secondary psychiatric PDs, while miscellaneous disorders comprised dermatoses caused by psychotropic medications or substance use disorders [2,15,17,18]. Figure 1 outlines the algorithm for the classification of PD in this study.

All the study participants gave both written and verbal informed consent before being recruited into the study. Patients with mental health disorders with chronic debilitating systemic conditions such as uncontrolled diabetes mellitus, chronic renal disease, advanced heart failure, retroviral disease, and malignancies were excluded from the study. Unstable mental health state and pregnancy were additional exclusion criteria.

### Sociodemographic and Clinical Characteristics of the Study Population

A data pro forma administered by the researchers was used to document relevant information, including sociodemographic, medical, and drug history, from all the participants. Information regarding the underlying mental health diagnosis and treatment was obtained from the patients’ medical records. A whole-body clinical examination for skin conditions was performed on all the recruited participants, and clinical findings were documented in the data pro forma. The diagnosis of skin conditions was mostly clinical. To support or confirm the clinical diagnosis, investigations such as skin scraping for fungal studies, woods lamp examination, and skin biopsy for histology were performed when needed.

### Prevalence of PDs and Association With Psychiatric Conditions

The participants were categorized into 3 groups based on their psychiatric diagnosis: schizophrenia; affective disorders; and anxiety, stress-related, and somatoform disorders. Psychodermatological dermatoses were noted for each patient within their respective diagnostic groups to determine prevalence, and comparisons were made between groups using appropriate statistical tests of association.

### Data Analysis

Data entry and analysis were performed using SPSS software (version 21; IBM Corp). A univariate analysis was performed to summarize the demographic characteristics of the participants. Categorical variables of interest were recoded as dichotomous variables (with exception of level of education) and analyzed using bivariate statistical tests. The Fisher exact test and likelihood ratio were used in place of the chi-square test in cases where the chi-square assumption was violated. Symmetry and measure of association between the variables were performed using phi and odds ratio, respectively. The level of statistical significance was set at .05.

### Ethical Considerations

This study was carried out in accordance with the Declaration of Helsinki and was approved by the Ethics and Research Committee of the Obafemi Awolowo University Teaching Hospitals Complex (national and international registration numbers NHREC/27/02/2009a and IRC/IEC/00045553, respectively) before the commencement of the study.

## Results

### Sociodemographic and Clinical Characteristics of the Study Population

A total of 107 patients with mental health disorders participated in the study. The major categories of psychiatric diagnosis in the study population were schizophrenia (66/107, 61.7%), anxiety and related disorders (21/107, 19.6%), and affective disorders (20/107, 18.7%). Comorbid substance use disorders were diagnosed in 13 (12.1%) participants and were most prevalent in patients with affective disorders. Females outnumbered males by a ratio of 1.49:1. These are presented in Table 1.

There was no significant age or gender disparity among the various categories of psychiatric conditions. However, patients with schizophrenia were significantly less well-educated compared with other groups of psychiatric conditions (*P*=.04). Patients with schizophrenia were also significantly more likely to be treated with antipsychotic drugs compared to those with affective disorders, anxiety disorders, or somatoform disorders.

**Table 1:** Comparison of sociodemographic variables and clinical characteristics of the different groups of psychiatric conditions.

Demographics		Schizophrenia (n=66)	Affective disorders (n=20)	Anxiety, stress-related, and somatoform disorders (n=21)	*P* value
Age (years), mean (SD)		39.52 (11.62)	39.3 (13.74)	45.91 (15.9)	.13
**Sex, n (%)**					.32
	Female	42 (64)	9 (45)	13 (62)	
	Male	24 (36)	11 (55)	8 (38)	
**Marital status, n (%)**					.005
	Single	37 (56)	11 (55)	5 (24)	
	Married	18 (27)	9 (45)	13 (62)	
	Separated, divorced, and widowed	11 (17)	—^a^	3 (14)	
**Education, n (%)**					.04
	No formal education	3 (4)	—	—	
	Primary	9 (14)	5 (25)	4 (19)	
	Secondary	32 (48)	3 (15)	6 (29)	
	Tertiary	22 (33)	12 (60)	11 (52)	
**Comorbid substance use disorder, n (%)**					.87
	No	58 (88)	17 (85)	19 (90)	
	Yes	8 (12)	3 (15)	2 (10)	
**Type of treatment, n (%)**					<.001
	Nonpharmacological	1 (2)	5 (25)	11 (52)	
	Antipsychotics	65 (98)	15 (75)	10 (48)	
**Dermatological disorder, n (%)**					
	Psychodermatological disorder	45 (68)	15 (75)	15 (71)	.83
	Reported or sought treatment for skin conditions	12 (27)	9 (60)	11 (73)	.002

^a^Not applicable.

### Prevalence of PDs and Association With Psychiatric Conditions

The pattern and distribution of PDs are presented in Table 2, while Table 3 shows the association between PDs and primary psychiatric conditions in the study population. A total of 75 (70.1%) out of 107 participants had at least one comorbid PD. Although the proportion of patients with PDDPCs (47/107, 43.9%) exceeded that with PPDDMs (42/107, 39.3%), PPDDMs were the overall most frequently diagnosed group of PDs, accounting for 61 of the 129 (47.3%) different psychodermatological diagnoses in the study population. Neurotic excoriation, followed by psychogenic pruritus and onychophagia, were the 3 most frequent PPDDMs in this study, while delusion of parasitosis (5/107, 4.7%) was among the least frequent.

The total prevalence of PDs did not differ significantly between groups of psychiatric conditions. However, significant associations were found between some psychiatric conditions and comorbid PDs. Compared to patients with either schizophrenia or affective disorders, patients with anxiety disorders were significantly more likely to be diagnosed with PPDDMs resulting from delusions or hallucinations and somatoform disorders (*P*=.01; odds ratio [OR] 9.88, 95% CI 1.67-58.34 and *P*=.003; OR 13.13, 95% CI 2.34-73.65), respectively*.* They were also significantly less likely to be diagnosed with PDDPCs (OR 0.24, 95% CI 0.07-0.76), particularly psychophysiological dermatoses (OR 0.18, 95% CI 0.04-0.81)*.* On the other hand, patients with schizophrenia demonstrated a moderate negative association with a diagnosis of PPDDMs resulting from delusions or hallucinations (*P*=.002; ϕ=–0.309). Following binary regression analysis, female sex was the only significant predictor of both PPDDM and PDDPC, while obesity was predictive of PDDPCs (Table S4 in Multimedia Appendix 1).

**Table 2:** Relative frequency and distribution of psychodermatological disorders (PDs) among patients with psychiatric conditions.

				Schizophrenia (n=45), n (%)		Affective disorder (n=15), n (%)		Anxiety, stress-related, and somatoform disorders (n=15), n (%)	Total (n=75), n (%)
**Primary dermatological disorders with psychiatric comorbidities**									
	**Psychophysiological dermatoses**								
		Seborrheic dermatitis	11 (24)		5 (33)		1 (6)		17 (22)
		Acne vulgaris	11 (24)		4 (26)		1 (6)		16 (21)
		Atopic dermatitis	0 (0)		1 (6)		0 (0)		1 (1)
		Hyperhidrosis	1 (2)		0 (0)		0 (0)		1 (1)
		Urticaria	1 (2)		0 (0)		0 (0)		1 (1)
	**Secondary psychiatric PDs**								
		Alopecia	9 (20)		2 (13)		1 (6)		12 (16)
		Ichthyosis	4 (8)		0 (0)		0 (0)		4 (5)
		Vitiligo	2 (4)		1 (6)		1 (6)		4 (5)
		Other chronic eczemas	1 (2)		2 (13)		0 (0)		3 (4)
**Primary psychiatric disorders with dermatologic manifestations**									
	**Self-inflicted dermatoses**								
		Neurotic excoriation	11 (24)		2 (13)		4 (26)		17 (22)
		Onychophagia	6 (13)		0 (0)		1 (6)		7 (9)
		Acne excoriee	1 (2)		0 (0)		1 (6)		2 (2)
		Dermatitis artefacta	1 (2)		0 (0)		0 (0)		1 (1)
	**Disorders resulting from delusions or hallucinations**								
		Delusion of parasitosis	0 (0)		1 (6)		4 (26)		5 (6)
		Body dysmorphia	0 (0)		1 (6)		0 (0)		1 (1)
	**Somatoform disorders**								
		Cutaneous pain syndromes	2 (4)		0 (0)		4 (26)		6 (8)
		Venereophobia	0 (0)		0 (0)		1 (6)		1 (1)
	**Disorders resulting from compulsion**								
		Psychogenic pruritus	11 (24)		1 (6)		2 (13)		14 (18)
		Lichen simplex chronicus	3 (6)		2 (13)		2 (13)		7 (9)
**Miscellaneous PDs**									
	Cutaneous stigmata of substance use disorders		3 (6)		0 (0)		1 (6)		4 (5)
	Antipsychotic induced pigmentary changes		3 (6)		1 (6)		0 (0)		4 (5)
	Other cutaneous adverse drug reactions		0 (0)		1 (6)		0 (0)		1 (1)

**Table 3:** Association between psychodermatological disorders and primary psychiatric conditions.

Psychodermatological disorder			Affective disorders (n=20)							Anxiety, stress-related, and somatoform disorders (n=21)							Schizophrenia (n=66)				
			Patients, n (%)		*P* value		OR^a^ (95% CI)		Patients, n (%)			*P* value		OR (95% CI)		Patients, n (%)			*P* value		OR (95% CI)
**PDDPC^b^**			11 (55)		.26		1.73 (0.65-4.61)		4 (19)			.01		0.24 (0.07-0.76)		32 (48)			.23		1.63 (0.73-3.62)
	Psychophysiological dermatoses	9 (45)		.16		2.03 (0.750-5.99)		2 (10)			.02		0.18 (0.04-0.81)		23 (35)			.39		1.46 (0.62-3.44)	
	Secondary psychiatric	5 (25)		.44		1.28 (0.410-3.99)		2 (10)			.11		0.33 (0.07-1.52)		16 (24)			.39		1.55 (0.58-4.18)	
**PPDDM^c^**			5 (25)		.15		0.45 (0.150-1.35)		12 (57)			.06		2.49 (0.94-6.58)		25 (38)			.71		0.86 (0.39-1.91)
	Self-inflicted dermatoses	2 (10)		.11		0.33 (0.070-1.53)		4 (19)			.47		0.78 (0.23-2.58)		18 (27)			.13		2.19 (0.79-6.08)	
	Dermatoses resulting from delusions or hallucinations	2 (10)		.31		2.31 (0.392-13.57)		4 (19)			.01		9.88 (1.67-58.34)		—^d^			.01		0.04 (0.00-0.75)	
	Somatoform disorders	—		.22		0.26 (0.014-4.78)		5 (24)			.003		13.13 (2.34-73.65)		2 (3)			.07		0.23 (0.04-1.22)	
	Dermatoses resulting from compulsion	3 (15)		.41		0.68 (0.178-2.56)		4 (19)			.61		0.96 (0.28-3.21)		14 (21)			.60		1.31 (0.48-3.57)	
**Miscellaneous psychodermatological disorders**			2 (10)		.46		1.50 (0.280-8.05)		1 (5)			.51		0.56 (0.07-4.85)		5 (8)			.64		1.04 (0.24-4.56)
	Adverse effects of medications	1 (5)		.65		1.09 (0.115-10.33)		—			.33		0.34 (0.02-6.48)		4 (6)			.36		2.58 (0.28-23.93)	
	Cutaneous manifestation of substance use disorders	—		.43		0.45 (0.023-8.766)		1 (5)			.59		1.38 (0.14-14.01)		3 (5)			.50		1.91 (0.19-18.95)	

^a^OR: odds ratio.

^b^PDDPC: primary dermatological disorder with psychiatric comorbidity.

^c^PPDDM: primary psychiatric disorder with dermatologic manifestations.

^d^Not available.

## Discussion

### Background

This study demonstrates a high burden of PDs among patients with mental health disorders. There are significant gaps in the scientific literature on PDs in the context of primary psychiatric conditions. Most data on PDs are derived from the general population or dermatology outpatient settings and have consistently demonstrated a high prevalence of comorbid psychiatric symptoms among patients with diverse dermatological conditions [5-7,19-21].

### Prevalence of PDs

We recorded a high (75/107, 70%) prevalence of PDs in this study, with more than half (54/107, 50.46%) of the population having multiple PDs. The exact prevalence of PDs in those with primary psychiatric conditions is uncertain due to limited research, a lack of standard classification system, and underdiagnosis [18,22]. Nevertheless, previous studies highlight the significant burden of psychiatric problems among patients with dermatological conditions [21-23]. Our findings echo this, underscoring the substantial relationship between dermatological and psychiatric conditions.

### Pattern of PDs in Patients With Mental Health Disorders

PDDPCs are commonly diagnosed noninfectious dermatoses in patients with mental health disorders [24-26]. This study found that 43.9% (47/107) of patients had at least one PDDPC. Comparable rates were reported among patients with dermatological conditions seeking psychodermatological consultations in Singapore (31.6%) [27] and patients with psychiatric conditions in Morocco (34.3%) [18]. The most frequent PDDPC was seborrheic dermatitis, affecting 17 of 107 (15.9%) patients. This is consistent with earlier reports of a high prevalence of seborrheic dermatitis in patients with mental health disorders [18,24] and may be linked to seborrhea induced by psychotropic medications.

The prevalence and pattern of secondary psychiatric conditions in this study mirror observations among patients with mental health disorders in Morocco, where alopecia and vitiligo were the most frequent secondary PDDPCs [18]. A similar prevalence of hair loss (14.9%) was also reported among patients with mental health disorders in India [28]. In contrast to research conducted among patients with dermatological conditions [29], the incidence of alopecia seems remarkably higher in patients with mental health disorders. This may be related to the impact of psychological stress and psychotropic medications on hair growth [30,31].

PPDDMs comprise dermatoses whose complex etiology is rooted in pathological psychological processes. The significant association between PPDDMs and primary psychiatric conditions is corroborated by our research findings, in which more than a third (42/107, 39.3%) of patients with mental health disorders were diagnosed with PPDDMs. A comparable prevalence of PPDDMs (22%) among patients with mental health disorders was reported by Barrimi et al [18] in Morocco. However, lower rates have also been documented by various authors, ranging from 5.4% among patients with mental health disorders in Nepal [32] to 8.7% in an Egyptian study [25]. Differences in the nomenclature and categorization systems adopted by various authors researching PDs may be responsible for these variations.

The least common PDs in this study were miscellaneous PDs, primarily encompassing cutaneous reactions to psychotropic drugs and skin signs of substance use disorders, which both occurred in 4% (4/107) of the study population. This corresponds with the 2%-5% prevalence documented for adverse reactions to psychiatric medications in the literature [33-35]. However, skin manifestations of substance abuse are rarely mentioned in studies documenting skin conditions in patients with mental health disorders [32,36].

### Relationship Between Psychiatric Conditions and PDs

#### Affective Disorders and PDs

The prevalence of PDs, especially PDDPCs, was highest in patients with affective disorders compared with other psychiatric conditions (Table S5 in Multimedia Appendix 2). While this contrast lacked statistical significance in this study, previous investigators have reported a significant association between PDDPCs, specifically psychophysiological disorders, and affective disorders [6,24,37,38]. The pathophysiological mechanism connecting PDs and affective disorders may be related to disrupted allostasis [39]. This occurs when persistent psychological and physical stress from long-standing skin conditions overwhelms normal homeostatic mechanisms and initiates pathological processes that are detrimental to mental health. Conversely, persistent psychological stress and emotional conflicts can trigger neuroimmunological and hormonal mediators capable of inducing pathological changes in the skin [40,41]. In either situation, a vicious chain of events that is characteristic of psychophysiological disorders is established, in which skin conditions cause psychological distress, which in turn exacerbates preexisting dermatoses.

#### Anxiety, Stress-Related, and Somatoform Disorders

In this study, patients with anxiety, stress-related, and somatoform disorders had the lowest prevalence of PDDPCs and were less likely to be diagnosed with psychophysiological dermatoses. (ϕ=–0.236; *P*=.02). This contrasts with dermatology outpatient findings that often associate anxiety disorders with PDs [23]. Possible explanations for the negative association between anxiety disorders and PDDPCs in this study could be the influence of psychotropic medications. Furthermore, the heightened inclination of patients with anxiety disorder in this study to seek treatment for their skin complaints could have played a role in the reduced prevalence and negative correlation of anxiety with PDDPCs.

PPDDMs resulting from delusions, hallucinations, and somatoform disorders, unlike PDDPCs, showed a strong association with anxiety disorders. Patients with anxiety disorders were 13 times more likely to be diagnosed with somatoform disorders and 9 times more likely to have PPDDMs from delusions or hallucinations, compared to schizophrenia or affective disorders. Notably, 4 (80%) out of 5 cases of delusion of parasitosis and 5 out of 7 cases of somatoform disorders were identified in patients with anxiety disorders. Although delusions of parasitosis have been previously linked to anxiety disorders [42], most researchers have presented differing associations with various psychiatric conditions [36,43,44]. Consequently, the exact relationship between delusional parasitosis and specific comorbid psychiatric conditions remains controversial. Further studies are required to better understand the conditions in relation to underlying psychiatric conditions.

#### Schizophrenia and PDs

Patients with schizophrenia had the lowest prevalence of PDs. However, self-inflicted dermatoses like neurotic excoriations and secondary psychiatric dermatoses such as alopecia were more common in patients with schizophrenia than in those with other psychiatric conditions. Reports of self-inflicted dermatoses in patients with schizophrenia are uncommon [45-47]. Rather, most studies demonstrate an association between self-inflicted dermatoses and major depression, obsessive-compulsive disorder (OCD), and anxiety disorders [46,48-50]. The higher prevalence of self-inflicted dermatoses among patients with schizophrenia in this study, therefore, contradicts popular findings in the literature. However, considering the recent reclassification of self-inflicted dermatoses within the OCD spectrum [51] and previous reports of high comorbidity of OCD with schizophrenia [52,53], our findings may indicate a potential underdiagnosis of coexisting OCD in the group of patients with schizophrenia.

In this study, schizophrenia had a significant negative relationship with delusion of parasitosis (ϕ=–0.309; *P*=.001), which is at variance with earlier reports [36,54-56]. This could be due to the effects of antipsychotics on delusional symptoms because, compared to the cohort of patients with the highest frequency of delusion of parasitosis (patients with anxiety disorder) in this study, patients with schizophrenia were significantly more likely to be treated with antipsychotics *(P*<.001).

### Conclusions

This study provides important insights into the overwhelming burden of psychodermatological conditions in patients with mental health disorders and specific associations with underlying psychiatric diagnosis. Noteworthy is the significant association between anxiety disorders and PPDDMs due to delusions or hallucinations and somatoform disorders. The relationship between PDs and psychiatric conditions in patients with mental health disorders differs from reports of studies conducted among patients with dermatological conditions. Larger studies conducted among patients with mental health disorders are, however, required to confirm these findings.

### Study Limitations

There are some limitations to this study that can affect the degree to which our findings can be extrapolated to the general population. First is the relatively small sample size and relatively lower proportions of patients with anxiety and affective disorders. Second is the lack of representation of some other major categories of psychiatric conditions in the study population, and finally, the lack of a control group comprising patients without mental health disorders to enable the comparison of the burden and pattern of PDs between patients with and without psychiatric conditions.

## Appendix

Multimedia Appendix 1 Logistic regression analysis for predictors of Psychodermatological Disorders.

Multimedia Appendix 2 Comparison of the frequency and pattern of psychodermatological disorders among the major groups of major psychiatric conditions.

## Abbreviations

DSM-IV: Diagnostic and Statistical Manual of Mental Disorders, 4th edition
OCD: obsessive-compulsive disorder
OR: odds ratio
PD: psychodermatological disorder
PDDPC: primary dermatological disorder with psychiatric comorbidity
PPDDM: primary psychiatric disorder with dermatologic manifestations
