# Supplementary material for: Psychodermatological Disorders in Patients With Primary Psychiatric Conditions: Cross-Sectional Study
Source: JMIR Dermatol. 2023 Oct 2;6:e47769. doi: 10.2196/47769 (PMC10580141; doi:10.2196/47769)
Supplement: Multimedia Appendix 1 [file derma_v6i1e47769_app1.docx]

**Multimedia Appendix 1**

**Table S4.** Logistic regression analysis for the predictors of primary psychiatric disorders with dermatological manifestations and primary dermatologic disorders with psychiatric comorbidities.

|  | Primary psychiatric disorders with dermatologic manifestations | | | Primary dermatologic disorders with psychiatric co-morbidities | | |
| --- | --- | --- | --- | --- | --- | --- |
| **Independent Variables** | aOR^a^ | 95% CI for aOR^a^ | P value | aOR^a^ | 95% CI for aOR^a^ | P value |
| **Sex** |  |  |  |  |  |  |
| Female (Ref) |  |  |  |  |  |  |
| Male | 0.30 | 0.12 - 0.77 | .01 | 0.37 | 0.15 -0 .91 | .03 |
| **Age** |  |  |  |  |  |  |
| 18 -44 years( Ref) |  |  |  |  |  |  |
| 45 and above | 0.59 | 0.23 -1.51 | .27 | 1.04 | 0.42 – 2.55 | .94 |
| **Body Mass Index** |  |  |  |  |  |  |
| Normal BMI (Ref) |  |  |  |  |  |  |
| Overweight or Obese | 1.68 | 0.67 – 4.21 | .27 | 0.39 | 0.15 -0.98 | .04 |
| **Social Class** |  |  |  |  |  |  |
| Upper Social Class (Ref) |  |  |  |  |  |  |
| Lower Social Class | 1.63 | 0.53 – 5.06 | .39 | 0.79 | 0.28 – 2.25 | .65 |
| **Functional Status** |  |  |  |  |  |  |
| Independent (Ref) |  |  |  |  |  |  |
| Dependent | 0.62 | 0.25 -1.55 | .31 | 1.14 | 0.46 – 2.84 | .77 |
| **Psychiatric Diagnosis** |  |  |  |  |  |  |
| Schizophrenia | 0.35 | 0.11 – 1.05 | .06 | 2.43 | 0.56 – 10.6 | .24 |
| Affective Disorders | 0.41 | 0.06 - 2.57 | .34 | 4.47 | 0.77– 25.83 | .1 |
| Anxiety, Stress Related & Somatoform Disorders | 2.12 | 0.39 -1.63 | .39 | 0.52 | 0.09 – 3 | .47 |
| aOR^a^= adjusted Odds Ratio. | | | | | | |
