# Supplementary material for: Psychodermatological Disorders in Patients With Primary Psychiatric Conditions: Cross-Sectional Study
Source: JMIR Dermatol. 2023 Oct 2;6:e47769. doi: 10.2196/47769 (PMC10580141; doi:10.2196/47769)
Supplement: Multimedia Appendix 2 [file derma_v6i1e47769_app2.docx]

**Multimedia Appendix 2**

**Table S5. Comparison of the frequency and pattern of psychodermatological disorders among the major groups of psychiatric conditions.**

|  | Schizophrenia  n=66 (%) | Affective disorders n= 20 (%) | Anxiety, Stress Related and Somatoform disorders  n= 21(%) | STATISTICS  (Chi^2^) | *df* | *P value* |
| --- | --- | --- | --- | --- | --- | --- |
| Skin disease present | 59(89) | 18(90) | 18(86) | 0.44^a^ |  | .89 |
| Psychodermatological disorders | 45(68) | 15(75.) | 15(71) | 0.36 | 2 | .83 |
| Non-psychodermatological disorders | 47(71) | 13(65) | 13(62) | 0.76 | 2 | .69 |
|  |  |  |  |  |  |  |
| **PDDPC** | 32(47) | 11(55) | 4(19) | 6.83 | 2 | .03 |
| Psychophysiological Dermatoses | 23(35) | 9(45) | 2(10) | 6.7 | 2 | .04 |
| Secondary Psychiatric | 16(24) | 5(25) | 2(10) | 2.56 ^a^ | 2 | .28 |
| **PPDDM** | 25(38) | 5(25) | 12(57) | 4.58 | 2 | .1 |
| Self-inflicted (factitious disorders) | 18(27) | 2(10) | 4(19) | 3.11 ^a^ | 2 | .21 |
| Dermatoses resulting from delusions or Hallucinations | - | 2(10) | 4(19) | 12.78 ^a^ | 2 | .002 |
| Somatoform disorders | 2(3) | - | 5(24) | 10.73 ^a^ | 2 | .005 |
| Dermatoses resulting from compulsion | 14(21) | 3(15) | 4(19) | 0.40 ^a^ | 2 | 0.82 |
| **Miscellaneous PDs** | 5(8) | 2(10) | 1(5) | 0.42 ^a^ | 2 | 0.81 |
| Adverse effects of medications | 4(6) | 1(5) | - | 2.28 ^a^ | 2 | 0.32 |
| Cutaneous manifestation of substance use disorders | 3(4) | - | 1(5) | 1.69 ^a^ | 2 | 0.43 |
|  |  |  |  |  |  |  |
| PDDPC = Primary Dermatologic Disorder with Psychiatric Co-morbidity  PPDDM = Primary Psychiatric Disorder with Dermatologic Symptoms  PD= Psychodermatoses  ^a^ = Likelihood ratio | | | | | | |
